# Supplementary material for: Synchronous deglacial thermocline and deep-water ventilation in the eastern equatorial Pacific
Source: Nat Commun. 2017 Jan 23;8:14203. doi: 10.1038/ncomms14203 (PMC5264251; doi:10.1038/ncomms14203)
Supplement: Supplementary Information — Supplementary Figure and Supplementary Tables [file ncomms14203-s1.pdf]

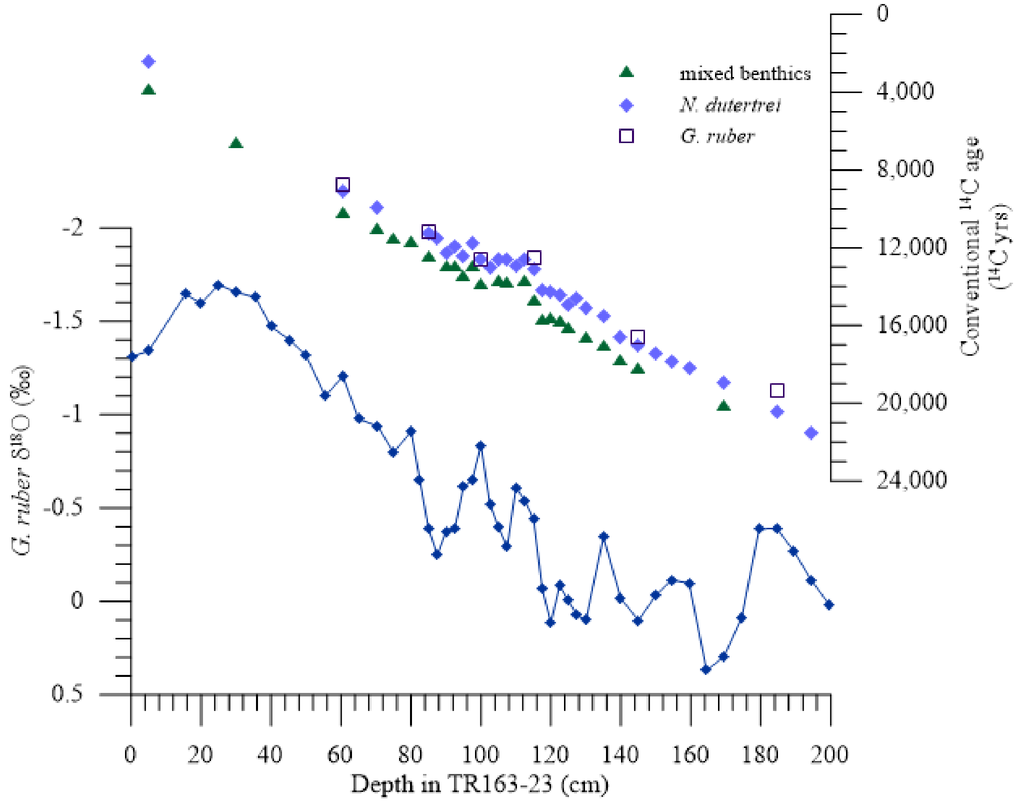

Supplementary Figure 1. TR163-23 *G. ruber* oxygen isotopes and conventional radiocarbon ages from benthic and planktonic foraminifera plotted versus core depth.

TR163-23 depth (cm)	Planktonic radiocarbon				Benthic radiocarbon				
	UCIAMS ID	taxa	14C age (14C yrs)	±1σ	UCIAMS ID	14C age (14C yrs)	±1σ	B-P (14C yrs)	±1σ
5	142516	*N. dutertrei*	2440	25	143508	3905	25	1465	35
30					152087	6705	30		
60.5	152095	*N. dutertrei*	9090	20	143509	10275	40	1185	45
60.5	141004	*G. ruber*	8790	25					
70.5	141005	*N. dutertrei*	9920	25	143510	11070	40	1150	47
75					152088	11575	35		
80					152089	11745	45		
85	159392	*N. dutertrei*	11250	25	143511	12540	55	1290	60
85	141006	*G. ruber*	11210	30					
87.5	162945	*N. dutertrei*	11485	20					
90	144872	*N. dutertrei*	12270	35	144881	13055	40	785	53
92.5	162946	*N. dutertrei*	11945	20	152090	13020	60	1075	63
95	144873	*N. dutertrei*	12425	35	144882	13500	45	1075	57
97.5	147290	*N. dutertrei*	11760	35	147285	13040	50	1280	61
100	159393	*N. dutertrei*	12575	40	143512	13960	60	1385	72
100	142517	*G. ruber*	12595	30					
102.5	162947	*N. dutertrei*	12995	25					
105	144874	*N. dutertrei*	12605	35	144883	13770	45	1165	57
107.5	147291	*N. dutertrei*	12630	40	147286	13890	60	1260	72
110	142518	*N. dutertrei*	12925	40					
112.5	147292	*N. dutertrei*	12600	45	147287	13780	60	1180	75
115	159394	*N. dutertrei*	13095	35	144884	14760	60	1665	69
115	144875	*G. ruber*	12525	30					
117.5	147293	*N. dutertrei*	14160	50	147288	15750	80	1590	94
120	142519	*N. dutertrei*	14260	45	143513	15660	70	1400	83
122.5	162948	*N. dutertrei*	14430	25	152091	15830	60	1400	65
125	144876	*N. dutertrei*	14970	45	144885	16230	60	1260	75
127.5	162949	*N. dutertrei*	14605	30					
130	162950	*N. dutertrei*	15085	30	152092	16690	50	1605	58
135	147294	*N. dutertrei*	15505	50	147289	17070	80	1565	94
140	162951	*N. dutertrei*	16625	35	152093	17890	50	1265	61
145	152096	*N. dutertrei*	16990	60	143514	18310	90	1320	108
145	142520	*G. ruber, sacculifer*	16600	70					
150	162952	*N. dutertrei*	17410	35					
154.5	144877	*N. dutertrei*	17840	60					
159.5	162953	*N. dutertrei*	18175	40					
169.5	144878	*N. dutertrei*	18960	70	152094	20180	90	1220	114
184.5	159395	*N. dutertrei*	20470	90					
184.5	144879	*G. ruber, sacculifer*	19380	110					
194.5	162954	*N. dutertrei*	21500	70					

Supplementary Table 1. Benthic and planktonic radiocarbon data from TR163-23.

TR163-23	Constant reservoir age (ΔR=147±13)			
depth (cm)	min age (yrs BP)	max age (yrs BP)	mean age (yrs BP)	Sedimentation rate (cm/kyr)
0.5	1011	1801	1477	
5	1795	2095	1923	10.08
15.5	2729	4170	3372	7.25
20	3233	4854	3990	7.27
25	3855	5544	4681	7.24
30	4510	6277	5400	6.95
35.5	5309	7036	6182	7.04
40	5978	7604	6798	7.31
45.5	6743	8275	7534	7.47
50	7456	8797	8157	7.23
55.5	8353	9338	8916	7.24
60.5	9495	9731	9591	7.41
65	9812	10399	10084	9.13
70.5	10566	10927	10709	8.80
75	10945	11730	11305	7.56
80	11545	12330	11974	7.47
82.5	11920	12562	12304	7.58
85	12511	12719	12622	7.86
87.5	12717	12963	12836	11.69
90	12910	13400	13183	7.19
92.5	13204	13522	13343	15.66
95	13382	13728	13555	11.78
97.5	13489	13860	13694	17.96
100	13701	14010	13854	15.64
102.5	13818	14213	13975	20.76
105	13914	14341	14062	28.67
107.5	14010	14500	14179	21.40
110	14182	14700	14406	11.00
112.5	14378	14957	14668	9.54
115	14758	15326	15013	7.24
117.5	15804	16430	16179	2.14
120	16329	16762	16547	6.80
122.5	16634	17019	16839	8.56
125	16876	17404	17161	7.77
127.5	17094	17582	17349	13.25
130	17487	17877	17684	7.46
135	18056	18518	18256	8.75
140	19049	19517	19312	4.73
145	19630	20045	19840	9.48
150	20147	20518	20343	9.93
154.5	20620	21018	20819	9.47

159.5	21042	21522	21282	10.79
164.5	21487	22080	21787	9.89
169.5	22018	22509	22298	9.80
174.5	22491	23270	22869	8.75
179.5	23016	23828	23428	8.94
184.5	23700	24291	24004	8.69
189.5	24242	24997	24628	8.00
194.5	25004	25464	25253	8.01
199.5	25369	26237	25763	9.80

Supplementary Table 2. Age model constructed using a constant reservoir age.

TR163-23 depth (cm)	Elastically derived Tie Points		
	Greenland tuned	Hulu cave tuned	Suigetsu 14C-plateau tuned
5	*N. dutertrei* 14C	*N. dutertrei* 14C	*N. dutertrei* 14C
60.5	*N. dutertrei* 14C	*N. dutertrei* 14C	*N. dutertrei* 14C
70.5	*N. dutertrei* 14C	*N. dutertrei* 14C	*N. dutertrei* 14C
80	GICC05 11443±90yrs	Hulu speleothem H82 11352±111	
90			plateau YD top 12499±137
95			
97.5			plateau YD base 13259±105
98.5			Hiatus
100	GICC05 13464±63 yrs	Hulu speleothem H82 13359±59	Plateau 1a top 13512±96
107.5			
110	GICC05 14486±75 yrs	Hulu speleothem H82 14363±58	
115			Plateau 1 base 15032±83
117.5			Plateau 2a top 15285±89
120			
122.5		Hulu speleothem H82 15618±86	Plateau 2a/2b 15792±106
130			Plateau 2b base 16551±138
135			
159.5		Hulu speleothem MSD 19497±113	
164.5			
199.5	GICC05 23992±204 yrs	Hulu speleothem MSD 24161±217	GICC05 23992±204 yrs

Supplementary Table 3. Tie point constraint of TR163-23 independent age models.

TR163-23	Greenland tuned age				Hulu tuned age			
depth (cm)	min age (yrs BP)	max age (yrs BP)	mean age (yrs BP)	Sedimentation rate (cm/kyrs)	min age (yrs BP)	max age (yrs BP)	mean age (yrs BP)	Sedimentation rate (cm/kyrs)
0.5	1041	1823	1502		965	1832	1500	
5	1836	2145	1978	9.46	1834	2157	1981	9.36
15.5	2733	4211	3422	7.27	2720	4247	3430	7.24
20	3249	4936	4055	7.10	3255	4962	4063	7.11
25	3883	5694	4758	7.11	3851	5697	4759	7.18
30	4539	6409	5459	7.14	4515	6435	5469	7.05
35.5	5279	7148	6218	7.25	5312	7120	6226	7.26
40	5923	7711	6843	7.20	5933	7683	6855	7.16
45.5	6738	8369	7594	7.33	6730	8372	7604	7.34
50	7454	8869	8215	7.25	7460	8874	8228	7.21
55.5	8422	9401	8987	7.12	8394	9405	8996	7.16
60.5	9561	9798	9656	7.47	9558	9795	9657	7.56
65	9858	10440	10141	9.28	9858	10450	10143	9.26
70.5	10596	10871	10726	9.40	10583	10861	10720	9.55
75	10845	11290	11058	13.57	10822	11274	11031	14.45
80	11223	11655	11421	13.77	11153	11635	11372	14.68
82.5	11390	11983	11659	10.51	11314	11947	11602	10.84
85	11567	12287	11904	10.22	11486	12225	11841	10.46
87.5	11760	12559	12150	10.15	11684	12491	12086	10.22
90	11987	12801	12394	10.22	11905	12728	12326	10.40
92.5	12237	13024	12642	10.11	12160	12927	12564	10.49
95	12491	13225	12890	10.06	12436	13122	12807	10.30
97.5	12811	13404	13140	10.03	12723	13286	13042	10.62
100	13212	13574	13389	10.02	13109	13447	13281	10.46
102.5	13406	13914	13644	9.83	13305	13794	13532	9.96
105	13611	14192	13897	9.85	13499	14064	13784	9.94
107.5	13861	14414	14152	9.82	13746	14270	14029	10.19
110	14200	14606	14401	10.06	14109	14435	14275	10.16
112.5	14372	15029	14661	9.60	14289	14813	14530	9.82
115	14531	15411	14921	9.61	14479	15102	14782	9.92
117.5	14719	15757	15186	9.46	14696	15348	15030	10.09
120	14910	16079	15448	9.52	14951	15561	15280	9.98
122.5	15121	16400	15714	9.41	15304	15758	15533	9.88
125	15340	16701	15981	9.37	15501	16145	15793	9.60
127.5	15575	17005	16247	9.37	15683	16501	16053	9.64
130	15799	17315	16511	9.50	15883	16815	16320	9.35

135	16245	17875	17045	9.36	16315	17415	16851	9.42
140	16712	18458	17571	9.51	16796	17960	17378	9.48
145	17172	19022	18093	9.58	17313	18483	17907	9.46
150	17674	19540	18619	9.49	17851	18962	18434	9.49
154.5	18143	20053	19102	9.32	18392	19345	18909	9.46
159.5	18648	20568	19631	9.46	19093	19749	19427	9.66
164.5	19189	21091	20154	9.56	19541	20490	19984	8.97
169.5	19720	21593	20682	9.46	19987	21175	20548	8.86
174.5	20261	22076	21209	9.50	20464	21801	21110	8.91
179.5	20826	22571	21741	9.39	20984	22396	21679	8.78
184.5	21416	23073	22276	9.35	21516	22946	22246	8.82
189.5	21998	23545	22810	9.36	22080	23503	22812	8.84
194.5	22614	23999	23335	9.51	22674	24008	23381	8.78
199.5	23256	24418	23859	9.55	23303	24483	23943	8.90

Supplementary Table 4. TR163-23 oxygen isotope tuned age models.

TR163-23	Plateau tuned age			
depth (cm)	min age (yrs BP)	max age (yrs BP)	mean age (yrs BP)	Sedimentation rate (yrs BP)
0.5	1043	1818	1481	
5	1833	2150	1977	9.07
15.5	2741	4256	3423	7.26
20	3302	4933	4057	7.10
25	3894	5660	4742	7.30
30	4534	6360	5448	7.08
35.5	5285	7096	6213	7.19
40	5923	7689	6844	7.14
45.5	6696	8354	7585	7.42
50	7441	8862	8214	7.15
55.5	8370	9402	8974	7.24
60.5	9557	9799	9656	7.33
65	9860	10451	10148	9.15
70.5	10604	10913	10740	9.29
75	10864	11472	11140	11.25
80	11233	11943	11577	11.43
82.5	11426	12165	11796	11.42
85	11649	12363	12015	11.44
87.5	11889	12548	12231	11.57
90	12172	12723	12451	11.35
92.5	12393	12977	12687	10.62
95	12634	13185	12921	10.65
97.5	12913	13360	13151	10.86
100	13268	13740	13484	7.52
102.5	13439	14018	13705	11.29
105	13629	14315	13959	9.83
107.5	13862	14571	14212	9.89
110	14099	14789	14463	9.96
112.5	14385	14973	14711	10.09
115	14764	15137	14959	10.10
117.5	15026	15382	15202	10.26
120	15220	15695	15451	10.06
122.5	15483	15929	15703	9.92
125	15696	16253	15960	9.74
127.5	15911	16523	16211	9.94
130	16173	16770	16463	9.92
135	16555	17502	16995	9.39
140	16966	18166	17524	9.45

145	17408	18763	18056	9.40
150	17857	19370	18591	9.35
154.5	18278	19889	19063	9.53
159.5	18781	20422	19594	9.42
164.5	19292	20977	20129	9.36
169.5	19784	21492	20655	9.49
174.5	20326	22024	21189	9.38
179.5	20875	22530	21722	9.37
184.5	21422	23013	22254	9.41
189.5	21990	23503	22783	9.45
194.5	22597	23976	23320	9.30
199.5	23190	24370	23802	10.39

Supplementary Table 5. TR163-23 radiocarbon plateau tuned age models.

TR163-23 depth (cm)	GICC05-tuned mean age (yrs BP)	*N. dutertrei*								Mixed benthic							
		$\Delta^{14}C_0$ (‰)	+ 2σ	- 2σ	$\Delta^{14}C_{0\text{-atm}}$ (‰)	+ 2σ	- 2σ	Reservoir age (14C yrs)	±	$\Delta^{14}C_0$ (‰)	+ 2σ	- 2σ	$\Delta^{14}C_{0\text{-atm}}$ (‰)	+ 2σ	- 2σ	Reservoir age (14C yrs)	±
5	1980	-62.47	19.7	16.1	-50.62	20.0	16.1	413	77	-218.76	15.7	13.6	-206.91	15.3	13.8	1878	83
30	5451									-159.93	97.2	91.9	-229.04	99.1	92.9	1926	454
60.5	9656	37.29	18.3	12.2	-50.28	18.6	12.9	360	43	-104.98	15.6	11.6	-192.55	15.6	12.1	1545	62
70.5	10727	64.73	19.1	16.9	-58.77	20.3	16.5	426	73	-77.29	17.0	14.7	-200.79	17.3	14.8	1576	91
75	11058									-98.05	25.3	23.4	-243.83	27.3	26.2	1915	110
80	11424									-77.27	26.0	23.0	-227.59	29.0	24.8	1755	77
85	11885	40.38	49.6	41.8	-146.18	52.5	43.8	1036	121	-113.97	41.8	35.7	-300.53	44.2	37.6	2326	128
87.5	12116	40.94	51.3	47.4	-161.85	53.3	51.8	1154	126								
90	12357	-27.62	48.6	48.5	-239.40	48.0	52.0	1770	195	-118.15	42.9	42.6	-329.93	44.5	44.2	2555	203
92.5	12599	43.29	48.8	49.5	-171.46	50.1	50.3	1237	264	-87.38	42.2	46.2	-302.14	41.8	48.2	2312	262
95	12832	12.78	42.7	48.1	-197.67	44.5	48.3	1465	270	-114.07	35.3	43.6	-324.53	38.4	44.4	2540	271
97.5	13075	133.88	36.6	45.7	-69.27	36.5	47.3	511	186	-33.13	30.7	39.7	-236.29	30.8	40.8	1791	191
100	13317	55.87	23.7	23.4	-142.90	24.1	22.7	1020	122	-111.35	20.5	19.8	-310.12	20.4	19.7	2405	119
102.5	13561	33.40	34.3	29.8	-162.03	33.7	30.5	1160	155								
105	13810	118.65	40.8	37.8	-79.46	41.1	38.2	596	146	-32.37	36.2	34.0	-230.48	38.3	32.6	1761	153
107.5	14059	150.05	36.9	41.2	-54.90	38.9	42.6	409	140	-16.90	31.1	33.9	-221.86	32.3	35.6	1669	156
110	14301	142.43	29.2	28.0	-77.87	37.3	30.6	531	57								
112.5	14556	227.69	55.6	42.5	-23.45	58.9	47.4	119	71	59.97	48.3	37.3	-191.17	50.6	42.8	1299	86
115	14816	191.22	69.2	56.6	-79.54	67.9	59.1	472	166	-31.78	56.9	47.4	-302.54	58.1	49.0	2137	169
117.5	15075	77.22	73.0	60.0	-208.91	74.4	64.0	1381	207	-116.22	62.4	51.3	-402.36	64.0	53.6	2971	202
120	15334	98.24	84.6	73.0	-201.11	84.8	74.1	1311	236	-77.41	70.7	61.8	-376.76	72.8	62.7	2711	240
122.5	15592	110.35	96.4	80.7	-199.57	97.8	82.6	1283	266	-67.24	73.0	64.4	-377.16	76.7	66.8	2683	274
125	15864	72.22	95.2	83.4	-248.66	93.2	83.0	1642	290	-83.44	78.8	75.3	-404.31	81.3	76.6	2902	292
127.5	16132	158.86	106.0	93.5	-175.25	109.3	93.1	1095	300								
130	16401	126.96	106.9	97.5	-219.02	107.1	96.0	1388	310	-77.14	93.3	75.7	-423.12	96.6	78.4	2993	306
135	16945	140.98	121.2	111.5	-227.44	118.7	109.6	1450	332	-61.00	95.4	90.3	-429.42	100.6	93.6	3015	330
140	17484	57.69	116.3	110.5	-329.76	111.5	109.9	2172	414	-96.42	101.5	95.8	-483.87	99.8	97.5	3437	410
145	18030	76.56	122.7	123.8	-321.47	123.7	125.3	2098	487	-86.57	107.1	102.8	-484.60	103.7	101.7	3418	486
150	18574	88.94	115.2	128.3	-315.09	110.6	128.0	2057	509								
154.5	19067	94.25	131.7	127.0	-316.43	127.5	127.7	2048	503								
159.5	19608	118.87	126.9	123.5	-299.26	127.2	130.2	1935	471								
169.5	20684	152.35	128.7	140.3	-293.40	123.3	149.1	1854	439	-10.02	108.7	113.9	-454.13	59.1	56.1	3018	234
184.5	22295	157.91	110.1	121.5	-333.80	111.0	123.5	2071	449								
194.5	23360	157.89	95.1	104.4	-349.27	99.9	100.6	2154	330								

Supplementary Table 6. Estimates of TR163-23 $\Delta^{14}C_0$, $\Delta^{14}C_{0\text{-atm}}$, and reservoir ages for deep and thermocline depth waters using the GICC05-tuned age model.
